# Air Way Management in Mini‐Surgical Percutaneous Dilatational Tracheostomy Using Endotracheal Tube and Supraglottic Airway Devices (I‐Gel, Laryngeal Mask Airway, and LarySeal) Classical and Ultrasound Point of View: A Prospective Cohort Study

**DOI:** 10.1002/hsr2.71642

**Published:** 2025-12-13

**Authors:** Seyed MohammadReza Hashemian, Ameneh Jafari, Hamidreza Jamaati, Mehran Malekshoar, Majid Malekmohammad, Batoul Khoundabi, Hossein Sadegh

**Affiliations:** ^1^ Chronic Respiratory Diseases Research Center (CRDRC), National Research Institute of Tuberculosis and Lung Diseases (NRITLD) Shahid Beheshti University of Medical Sciences Tehran Iran; ^2^ Tracheal Diseases Research Center (TDRC), National Research Institute of Tuberculosis and Lung Diseases (NRITLD) Shahid Beheshti University of Medical Sciences Tehran Iran; ^3^ Iran Helal Institute of Applied‐Science and Technology, Red Crescent Society of Iran Tehran Iran; ^4^ Iran University of Medical Sciences Tehran Iran

**Keywords:** endotracheal tube, ICU, msPDT, percutaneous dilatational tracheostomy

## Abstract

**Background and Aims:**

Mini‐surgical percutaneous dilatational tracheostomy (msPDT) offers a streamlined alternative to conventional tracheostomy, yet the optimal airway management device for this procedure remains underexplored. This study compares the efficacy and safety of endotracheal tubes (ETTs) versus supraglottic airway devices (SADs: I‐gel, LMA, LarySeal) during msPDT.

**Methods:**

In this prospective cohort study, 464 mechanically ventilated patients underwent msPDT between 2015 and 2023. Patients were stratified into four groups: ETT (*n* = 90), I‐gel (*n* = 180), LMA (*n* = 180), and LarySeal (*n* = 14). Procedural duration, hemodynamic parameters, oxygenation, and complications were analyzed. Ultrasound imaging assessed tracheal alignment and device‐related anatomical changes.

**Results:**

Procedural time was shortest with LMA (1.8 ± 0.65 min) and I‐gel (1.9 ± 0.62 min), compared to ETT (2.2 ± 0.68 min) and LarySeal (2.0 ± 0.66 min). The LarySeal group exhibited a higher pneumothorax incidence (7.1% vs. 0%–2.2% in other groups, *p* = 0.079) and tracheal displacement on ultrasound. Hemodynamic stability and oxygenation were comparable across groups, though SpO₂ declines were more frequent with ETT (5.6% vs. ≤ 1.7% for SADs).

**Conclusion:**

I‐gel and LMA optimize msPDT efficiency and safety, while LarySeal's anatomical interference warrants caution. Ultrasound‐guided device selection enhances procedural precision, advocating for SADs as first‐line tools in msPDT.

## 1 Introduction

Tracheostomy is a vital procedure for critically ill patients requiring prolonged mechanical ventilation in intensive care units (ICUs) [1]. Since its introduction in 1957, percutaneous dilatational tracheostomy (PDT) has become a preferred alternative to traditional surgical tracheostomy (ST) due to its minimally invasive nature, reduced procedural time, and lower complication rates [2, 3]. The evolution of PDT has been further enhanced by bronchoscopic guidance and specialized dilational kits, solidifying its role as the gold standard for ICU patients [4, 5]. However, traditional PDT is not without limitations, particularly in cases involving anatomical abnormalities, obesity, or contraindications to bronchoscopy [6, 7, 8].

To address these challenges, the mini‐surgical PDT (msPDT, Hashemian method) was introduced in 2015 as a novel bedside approach [3]. This technique combines the principles of PDT with controlled tissue retraction and ultrasound guidance to minimize complications such as posterior tracheal wall injury. Unlike conventional PDT, msPDT eliminates the need for bronchoscopy, simplifying the procedure while maintaining safety. Despite its growing adoption, there remains a significant gap in the literature: no prior studies have compared the efficacy of msPDT when performed using endotracheal tubes (ETTs) versus supraglottic airway devices (SADs), such as the laryngeal mask airway (LMA), I‐gel, or LarySeal. During msPDT, the use of an ETT presents specific procedural challenges. The tube physically occupies the tracheal lumen, which can obstruct the optimal trajectory for the introducer needle and dilators and carries a risk of accidental puncture [2, 9]. Furthermore, the necessary manipulation and partial withdrawal of the ETT to position the cuff just below the vocal cords increases the risk of unintended extubation and subsequent loss of the airway. SADs circumvent these issues by securing the airway from outside the trachea, thereby providing an unobstructed path for the procedure while maintaining continuous ventilation. This makes SADs a particularly appropriate choice for msPDT in patients who do not require the high‐pressure ventilation seal provided by an ETT.

Supraglottic airway devices have revolutionized airway management in anesthesia and critical care [10]. First‐generation devices like the LMA and second‐generation innovations such as the I‐gel (featuring a non‐inflatable gel cuff) and LarySeal (with a curved tube and inflatable cuff) offer distinct advantages, including ease of insertion, reduced hemodynamic instability, and lower risk of airway trauma compared to ETTs [11, 12, 13, 14]. As summarized in Table 1, each airway device offers distinct pros and cons depending on clinical requirements and patient conditions. However, their utility in msPDT, particularly regarding anatomical alignment, procedural efficiency, and safety, remains unexplored.

**Table 1:** Comparison of airway management devices, including their characteristics, advantages, and disadvantages.

Device	Image	Characteristics	Pros	Cons
ETT (Endotracheal Tube)	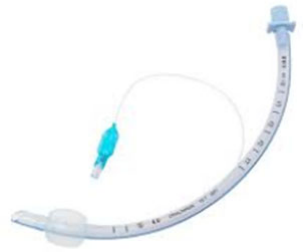	− Tube inserted via mouth/nose into trachea − Short‐to‐medium term − Invasive	− Gold standard for secure airway − Allows mechanical ventilation − Suction access	− Risk of laryngeal injury − Requires sedation − Discomfort during placement
LMA (Laryngeal Mask Airway)	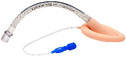	− Supraglottic device − Short‐term − Non‐invasive	− Easy/fast insertion − Minimal trauma − No laryngoscopy required	− Less secure (risk of aspiration) − Not for high airway pressures − Limited suction access
I‐Gel	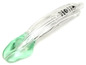	− Supraglottic with non‐inflatable gel cuff − Short‐term − Non‐invasive	− No cuff inflation needed − Better seal than LMA − Reduced gastric insufflation	− Displacement risk − Not for prolonged use − Limited suction access
Laryseal (Laryngeal Seal)	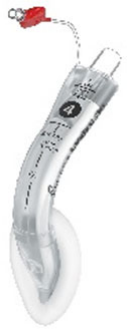	− Supraglottic with sealing mechanism − Short‐term − Non‐invasive	− Improved seal versus LMA − Reduced aspiration risk (varies) − Easy insertion	− Limited evidence/data − Not for high‐pressure ventilation − Brand‐specific variability
PDT (Percutaneous Dilational Tracheostomy)	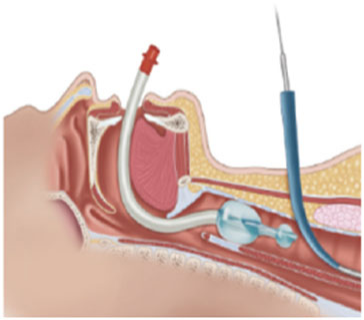	− Bedside tracheostomy via dilational technique − Medium‐to‐long term	− Faster than surgical tracheostomy − Less scarring − Lower infection risk	− Requires expertise − Risk of bleeding/stenosis − Contraindicated in obesity

*Note:* This study aimed to bridge this knowledge gap by evaluating clinical outcomes of msPDT using ETTs versus SADs. We focused on procedural duration, hemodynamic stability, and complications such as pneumothorax and subcutaneous emphysema. Additionally, we hypothesized that anatomical variations induced by device design (e.g., the curvature of the LarySeal) might impact procedural safety. By integrating ultrasound imaging to assess tracheal alignment, this study provides novel insights into the interplay between airway device selection and procedural success in msPDT.

## 2 Patients and Methods

### 2.1 Ethics Approval and Consent to Participate

This prospective cohort study received ethical approval from the Shahid Beheshti University of Medical Sciences (IRCT2015040120592N3) and was conducted at Masih Daneshvari Hospital between 2015 and 2023. Written informed consent was obtained from all participants or their legal representatives after a detailed explanation of the study's objectives.

### 2.2 Patients' Criteria

The study enrolled 464 critically ill adults aged 18–80 years who required mechanical ventilation for more than 7 days. Exclusion criteria included dissent from legal representatives, morbid obesity (BMI > 40), cervical spine trauma, enlarged thyroid, anatomical airway or tracheal abnormalities, ischemic heart disease (to avoid the potential for epinephrine‐induced tachycardia and hemodynamic instability, as well as stress‐induced myocardial ischemia during the procedure), or medical records predating 2015.

### 2.3 Sample Size

The sample size was determined based on procedural time as the primary outcome. A pilot study involving 20 patients revealed a standard deviation (SD) of 1.08 and a maximum allowable error (*E*) of 0.2. Using a Type I error rate (*α*) of 0.05 and statistical power (*β*) of 0.80, the calculated sample size was 457 as follows:

n=(2*〖(s)〗^2×〖(Z_α+Z_β)〗^2)/〖(E)〗^2=(2〖×(1.08)〗^2×〖(1.96+0.84)〗^2)/〖(0.2)〗^2≈457.

Attrition was handled by prospectively accounting for a 1.5% rate due to potential data loss, such as incomplete records or patient withdrawal post‐enrollment, which adjusted the initial sample size calculation from 457 to 464 patients. Due to the robust data registration system at our institution, there was no significant missing information in the data set.

### 2.4 Study Design and Patient Enrolment

**Figure 1:**
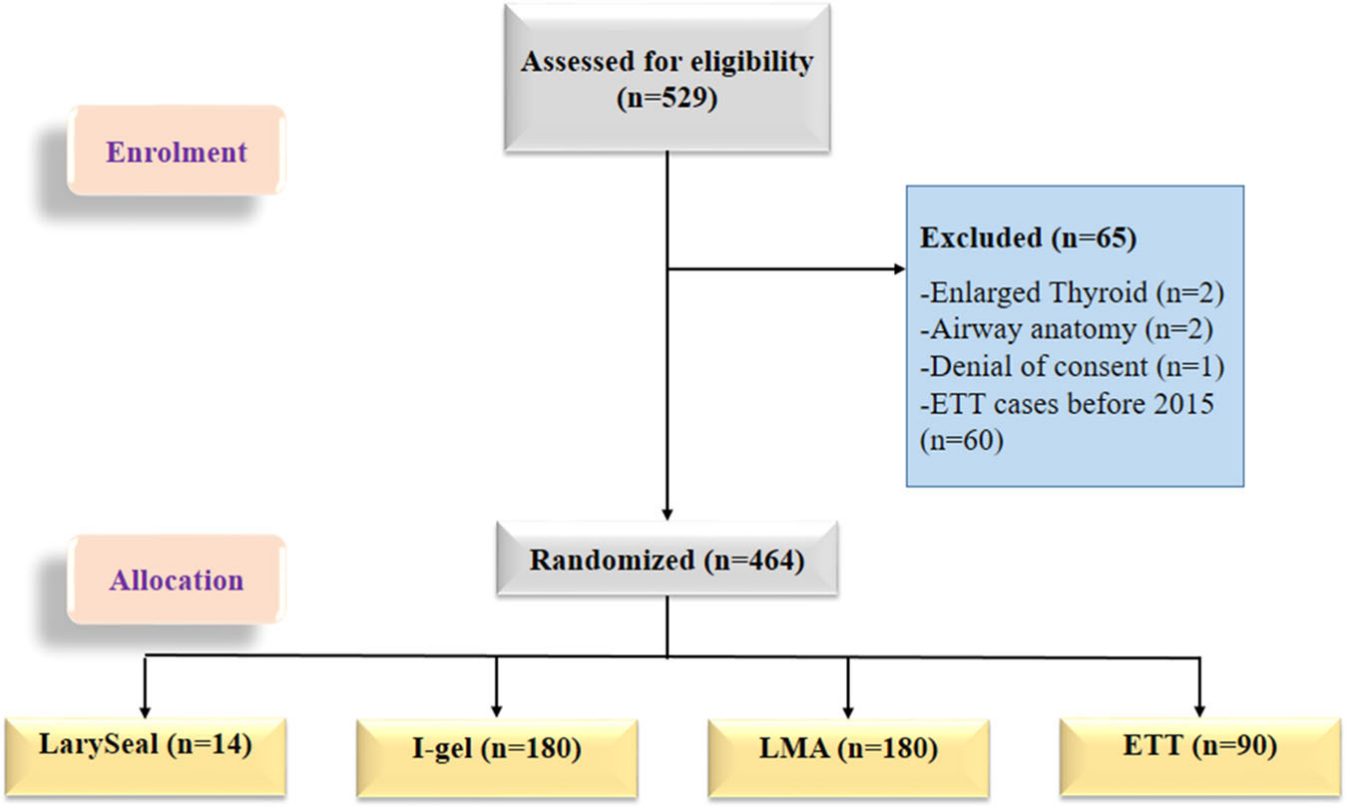
Consolidated Standards of Reporting Trials (CONSORT) diagram.

This was a prospective cohort study conducted between 2015 and 2023 at the Department of Critical Care, Masih Daneshvari Hospital, a university‐affiliated teaching hospital in Iran. The study included 464 patients who required tracheostomy and underwent msPDT. Patients were allocated into four independent groups based on the airway management device used during the procedure: endotracheal tube (ETT group, *n* = 90), laryngeal mask airway (LMA; Teleflex Medical, Dublin, Ireland) (LMA group, *n* = 180), I‐gel (Intersurgical Ltd., Wokingham, Berkshire, UK) (I‐gel group, *n* = 180), and LarySeal (Flexicare Medical Ltd., Mountain Ash, UK) (LarySeal group, *n* = 14) (Figure 1). Group allocation was determined by the attending intensivist's preference and clinical judgment, considering factors such as anticipated procedure difficulty, patient stability and device availability. All msPDT procedures were performed by a consistent team of experienced attending intensivists. Furthermore, all interventions adhered to a unified and standardized protocol, minimizing inter‐operator variability and ensuring a consistent level of expertise across all patient groups.

### 2.5 Patient Selection and Baseline Assessment

Inclusion criteria encompassed adult patients admitted to the intensive care unit (ICU) requiring prolonged mechanical ventilation and tracheostomy. Baseline and demographic characteristics were recorded, including sex, age, weight, ICU stay duration, mechanical ventilation time, Acute Physiology and Chronic Health Evaluation (APACHE) II scores, and underlying illnesses (e.g., amyotrophic lateral sclerosis [ALS], acute respiratory distress syndrome [ARDS]/H1N1, bronchiectasis, chronic obstructive pulmonary disease [COPD], COVID‐19/ARDS, cardiac arrest, multiple trauma, bacterial pneumonia).

### 2.6 Patient Preparation and Monitoring

Patients were fasted for at least 8 h prior to the procedure. Continuous monitoring included electrocardiography (ECG), pulse oximetry (SpO₂), non‐invasive blood pressure measurements, and end‐tidal carbon dioxide (ETCO₂) analysis. Vital signs, including mean arterial pressure (MAP), SpO₂, heart rate, and ETCO₂, were monitored and recorded throughout. Mechanical ventilation was adjusted to volume‐controlled mode with a tidal volume of 6 mL/kg. Sedation was achieved using midazolam (2–5 mg), analgesia with fentanyl (50–70 μg), and neuromuscular blockade with atracurium (0.5 mg/kg). Local anesthesia (2% lidocaine with 1/200000 epinephrine) was infiltrated at the incision site.

All patients were initially intubated with an ETT. Prior to the procedure, a comprehensive difficult airway assessment was performed, including evaluation of mouth opening, neck mobility, and tongue/airway edema. 100% oxygen was administered for at least 5 min. A difficult airway cart, including video laryngoscope, bronchoscope, supraglottic airway devices (SADs), bougies, and emergency kits for open cricothyrotomy, was readily available, along with trained staff. Lung ventilation was confirmed by auscultation, and SpO₂ and ETCO₂ monitoring.

### 2.7 Airway Management During msPDT

In the ETT group, the ETT cuff was deflated, and the tube was slowly retracted under direct laryngoscopy to position the cuff just below the vocal cords. The cuff was then re‐inflated to 20–30 cm H₂O to ensure no air leak, preventing needle puncture of the tube during the procedure.

**Figure 2:**
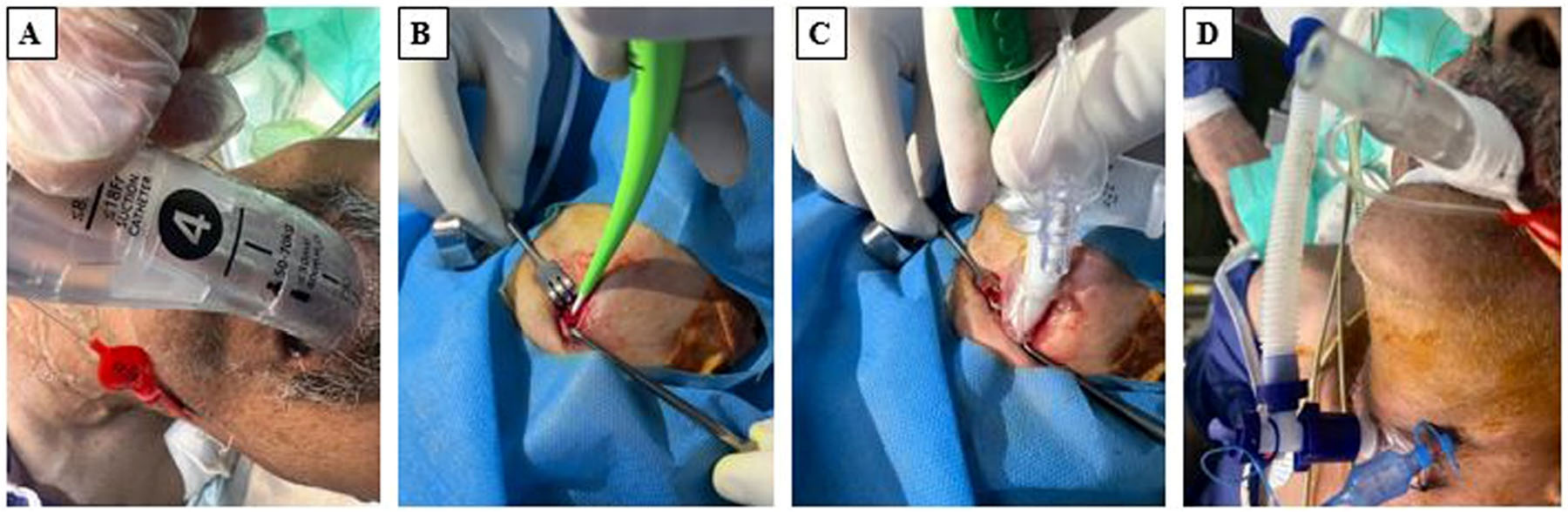
Insertion of supraglottic device (Laryseal) for airway management during msPDT (A), tissue dilator passing through the guide wire (B), introducing thracheostomy tube into dilated stoma (C), and transfering ventilator tube from supraglottic device to secured thrachestomy (D).

In the SAD groups (LMA, I‐gel, and LarySeal), patients were extubated, and the respective SAD was immediately inserted to maintain ventilation (Figure 2A). Cuff pressure for LMA and LarySeal was maintained at 40–50 cm H₂O to ensure optimal fit in the supraglottic space without air leak. The I‐gel, being cuffless, was positioned to achieve a secure seal based on manufacturer guidelines.

All procedures were performed by highly skilled intensivists experienced in msPDT, prioritizing safety in potential difficult airway scenarios.

### 2.8 msPDT Procedure

The patient was positioned supine with the neck extended. A 2‐cm vertical incision was made 1 cm below the cricoid cartilage. The trachea was punctured with an introducer needle at a 45° angle between the first and second or second and third tracheal rings, with placement confirmed by air bubble aspiration. Sequential dilation was performed (Figure 2B), followed by insertion of the tracheostomy tube (Figure 2C). Correct placement was verified via ETCO₂ detection and aspiration of tracheal secretions and the tracheostomy tube attached to the ventilator (Figure 2D).

### 2.9 Ultrasound Guidance

Ultrasound was used to assess tracheal anatomy, identify key landmarks (tracheal rings, cricoid cartilage, cricothyroid membrane, and thyroid cartilage), and evaluate airway device positioning during msPDT. This facilitated assessment of tracheal alignment and cartilage integrity after placement of ETT or SADs. A linear probe was positioned longitudinally in the midline of the neck to visualize the tracheal rings and guide accurate needle puncture. In the LarySeal group, ultrasound revealed a tracheal displacement of 3–6 mm.

### 2.10 Outcome Measures and Data Collection

Primary outcomes focused on procedural quality and safety, including procedural duration, procedural failure rate, number of tracheal puncture attempts, and intraoperative bleeding (quantified by the gauze pad weighing method [15]). Secondary outcomes included complications such as subcutaneous emphysema, pneumothorax, late bleeding (post‐procedure), and arrhythmia. All data were collected prospectively by trained research staff blinded to group allocation where possible.

### 2.11 Statistical Analysis

Statistical analysis was performed using IBM SPSS v26.0. The Kolmogorov‐Smirnov (K‐S) test was applied to check the normality of quantitative variables. Categorical variables such as sex, type of illness and complications were presented by frequency and percent (*n*%). Quantitative variables were showed by mean and standard deviation (SD), mean ± SD. To compare normal quantitative variables such as age and weight between the four study groups, the Analysis of Variance test (ANOVA) was used, and for non‐normal quantitative variables such as ICU time, Mechanical Ventilation time (MV), Procedure time and APACHE II, the non‐parametric Kruskal‐Wallis test was used. Examining the relationship between categorical variables was performed using Chi‐Square or Fisher's exact tests. Effect sizes such as Crammer coefficient for crosstabs tables, Eta squared (*η*2) for ANOVA and Kruskal‐Wallis, quantified the magnitude of an effect or relationship [16, 17]. The significance of all statistical tests was examined and reported at the 5% and in two tailed level.

## 3 Results

The study enrolled 529 critically ill patients requiring tracheostomy between 2015 and 2023, of whom 65 were excluded due to unmet inclusion criteria. The remaining 464 patients were allocated into four groups: ETT (*n* = 90), LMA (*n* = 180), I‐gel (*n* = 180), and LarySeal (*n* = 14).

Baseline demographic characteristics, including age, sex, weight, ICU stay duration, mechanical ventilation time, and APACHE II scores, showed no statistically significant differences between groups (*p* > 0.05) (Table 2).

**Table 2:** Baseline and demographic characteristics for patients.

Items	Group				*p* value	Effect size
	I‐GEL (*n* = 180)	LMA (*n* = 180)	LarySeal (*n* = 14)	ETT (*n* = 90)		
Sex/female (%)	109 (60.6)	111 (61.7)	9 (64.3)	55 (61.1)	0.70	0.09^a^
Age (years)	56.3 ± 14.2	50.4 ± 17.7	53.6 ± 15.9	56.0 ± 15.5	0.20	0.10^b^
Weight (Kg)	66.3 ± 12.1	69.4 ± 12.0	68.1 ± 11.8	68.4 ± 12.0	0.31	0.05^b^
ICU time (days)	16.9 ± 2.9	16.0 ± 3.2	16.8 ± 2.8	16.7 ± 2.9	0.36	0.05^b^
MV time (days)	9.7 ± 2.8	9.0 ± 2.6	9.5 ± 2.9	9.4 ± 2.8	0.21	0.04^b^
APACHE II	22.1 ± 2.7	22.6 ± 4.4	21.9 ± 3.0	22.0 ± 3.1	0.64	0.05^b^

^a^ Crammer effect size with degree of freedom (df) 3: Small: 0.01–0.17, Medium: 0.17–0.29, Large: equal or greater than 0.29.

^b^ Eta Square (*η*2) effect size: Small: 0.01–0.06, Medium: 0.06–0.14, Large: equal or greater than 0.14.

As summarized in Table 3, the majority of participants had diagnoses of chronic pulmonary diseases (e.g., COPD, bronchiectasis) or COVID‐19‐related ARDS, with no significant variation in illness distribution across groups (*p* > 0.05).

**Table 3:** Comparison type of illness between included patients.

Type of illness	Group				*p* value	Effect size^a^
	I‐GEL (*n* = 180) *N* (%)	LMA (*n* = 180) *N* (%)	LarySeal (*n* = 14) *N* (%)	ETT (*n* = 90) *N* (%)		
ALS	3 (1.7)	0 (0.0)	0 (0.0)	1 (1.1)	0.13	0.09
ARDS/H1N1	8 (4.4)	10 (5.6)	0 (0.0)	5 (5.5)	0.10	0.11
Bronchiectasis	21 (11.7)	18 (10.0)	1 (7.1)	9 (10.0)	0.58	0.12
COPD	35 (19.4)	30 (16.7)	2 (14.3)	16 (17.7)	0.41	0.09
ARDS/HIV	5 (2.8)	4 (2.2)	0 (0.0)	2 (2.2)	0.57	0.07
Cardiac arrest	3 (1.7)	0 (0.0)	0 (0.0)	2 (2.2)	0.11	0.12
Multiple trauma	5 (2.8)	3 (1.7)	1 (7.1)	2 (2.2)	0.36	0.11
COVID/ARDS	33 (18.3)	30 (16.7)	2 (14.3)	15 (16.6)	0.63	0.14
Bacterial Pneumonia	8 (4.4)	9 (5.0)	1 (7.1)	6 (6.7)	0.79	0.13

^a^ Crammer effect size with degree of freedom (df) 3: Small: 0.01–0.17, Medium: 0.17–0.29, Large: equal or greater than 0.29.

**Figure 3:**
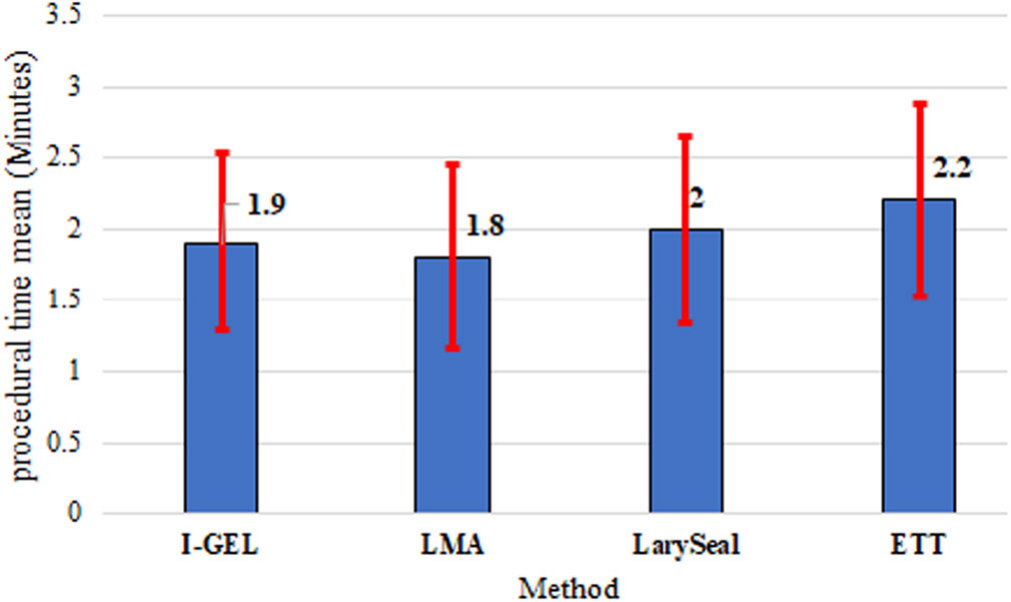
Error bars of procedure time by methods.

Procedural time was shortest in the LMA group (1.8 ± 0.65 min) and I‐gel group (1.9 ± 0.62 min), compared to the ETT group (2.2 ± 0.68 min) and LarySeal group (2.0 ± 0.66 min), however the difference was not significance (*p* = 0.12) and effect size equals to 0.09 with medium association, a trend toward faster completion with supraglottic devices was evident (Figure 3). Hemodynamic parameters, including mean arterial pressure (MAP), heart rate, and ETCO₂, remained stable across all groups before, during, and after the procedure, with no significant intergroup differences (*p* > 0.05). Oxygen saturation (SpO₂) declined slightly during and after the procedure in all groups, but this decline was most pronounced in the ETT group, where 5.6% of patients experienced SpO₂ drops compared to 1.7% in the I‐gel and LMA groups (*p* = 0.087).

Complications such as subcutaneous emphysema, pneumothorax, and arrhythmia were rare overall (Table 4). However, the LarySeal group exhibited a higher incidence of pneumothorax (7.1%) compared to the ETT (2.2%), LMA (0%), and I‐gel (0%) groups, though this difference did not reach statistical significance (*p* = 0.079). Intraoperative bleeding was minimal (< 2 cc in 97% of cases), with no significant variation between groups (*p* = 0.503). Procedural failures were absent in all groups except for one case in the ETT group (1.1%).

**Table 4:** The quality and complications.

Items		Group				*p*‐value	Effect size
		I‐GEL, *N* (%)	LMA, *N* (%)	LarySeal, *N* (%)	ETT, *N* (%)		
Bleeding during procedures	< 1.0 cc	175 (97.2)	174 (96.7)	13 (92.9)	85 (94.4)	0.50	0.12^a^
	1.0–2.0 cc	4 (2.2)	4 (2.2)	0 (0.0)	3 (3.4)		
	> 2.0 cc	1 (0.6)	2 (1.1)	1 (7.1)	2 (2.2)		
Multiple tracheal puncture		4 (2.2)	5 (2.8)	1 (7.1)	5 (5.6)	0.13	0.20^a^
Procedure fails		0 (0.0)	0 (0.0)	0 (0.0)	1 (1.1)	0.91	0.07^a^
Subcutaneous emphysema		1 (0.6)	1 (0.6)	0 (0.0)	3 (3.4)	0.44	0.10^a^
Pneumothorax		0 (0.0)	0 (0.0)	1 (7.1)	2 (2.2)	0.08	0.19^a^
Arrhythmia		4 (2.2)	3 (1.7)	0 (0.0)	3 (3.4)	0.62	0.10^a^
Late bleeding		1 (0.6)	1 (0.6)	0 (0.0)	2 (2.2)	0.78	0.08^a^
Procedure time (minutes)		1.9 ± 0.62	1.8 ± 0.65	2.0 ± 0.66	2.2 ± 0.68	0.12	0.09^b^

^a^ Crammer effect size with degree of freedom (df) 3: Small: 0.01–0.17, Medium: 0.17–0.29, Large: equal or greater than 0.29.

^b^ Eta Square (*η*2) effect size: Small: 0.01–0.06, Medium: 0.06–0.14, Large: equal or greater than 0.14.

**Figure 4:**
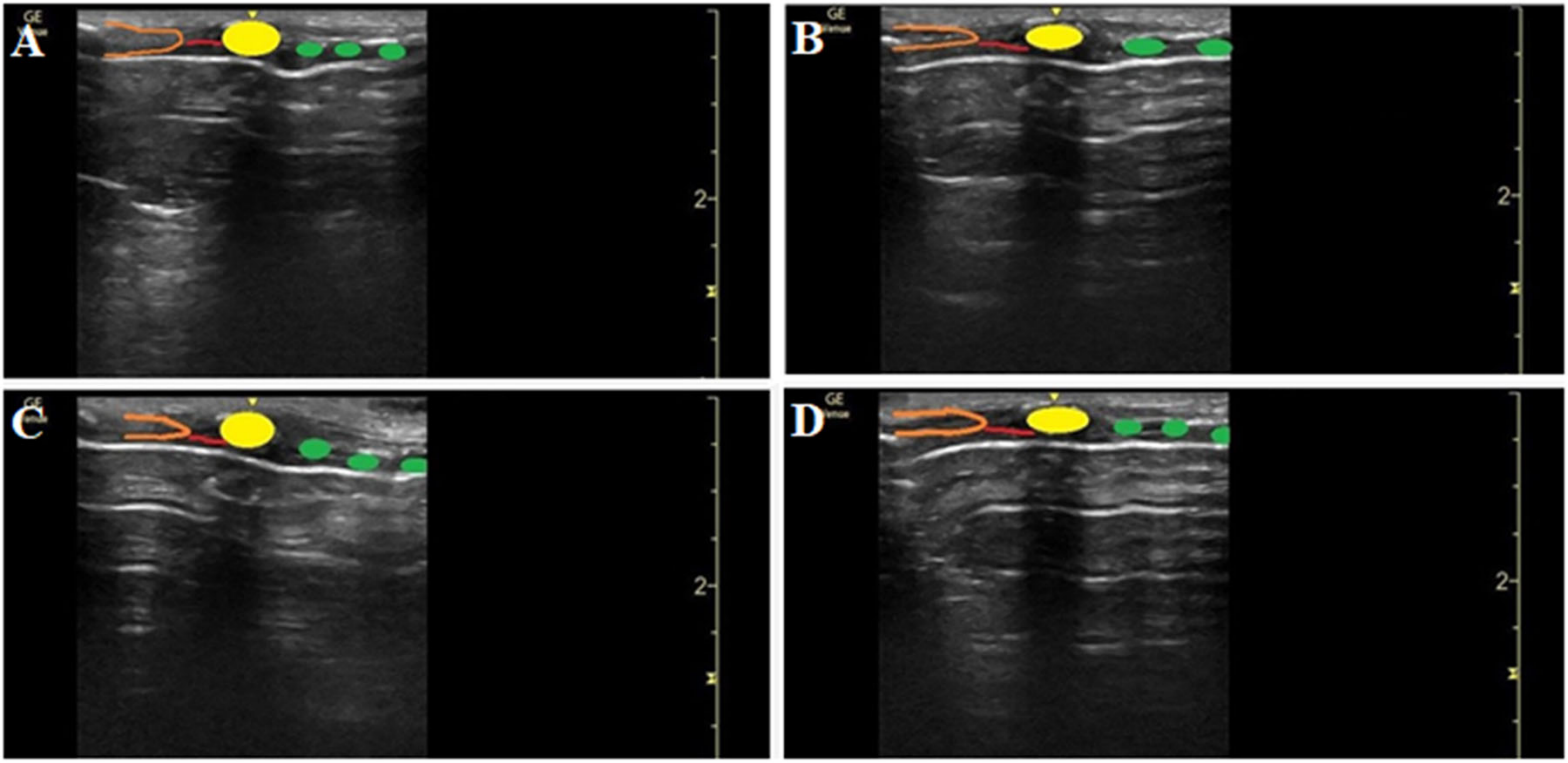
Longitudinal ultrasound view of the trachea while using ETT (A), I‐gel (B), LarySeal (C), and LMA (D). Longitudinal midline‐scan view of the neck shows the cricothyroid membrane in red. Orange, yellow, and green represent the thyroid cartilage, the cricoid cartilages, and tracheal rings, respectively.

Ultrasound imaging revealed that the LarySeal device caused anatomical displacement of the trachea, pushing tracheal rings deeper and altering alignment (Figure 4). This distortion was absent in the ETT, LMA, and I‐gel groups. The LarySeal's curvature correlated with increased procedural challenges, including repeated needle punctures and para‐tracheal insertion attempts, though these did not translate into statistically significant complication rates due to the small sample size.

## 4 Discussion

This study represents the first comparative analysis of airway management devices in msPDT. Our findings demonstrate that SADs, particularly the I‐gel and LMA, offer significant advantages over ETTs in procedural efficiency, with shorter insertion times and reduced airway manipulation.

Our findings align with existing literature supporting the use of SADs in various procedural contexts, however, it is important to acknowledge the limitations of these cited studies to better contextualize our conclusions. For instance, Panneer et al. [18] demonstrated advantages of the I‐gel over ETTs in elective cesarean sections, including faster insertion and improved hemodynamic stability, with no significant airway complications. The study also noted improved neonatal outcomes (e.g., higher 1‐min Apgar scores) and reduced maternal stress responses in the I‐gel group. These findings underscore the device's safety and efficacy in obstetric anesthesia, aligning with broader evidence favoring supraglottic airways for procedures requiring rapid airway management. The results further reinforce the applicability of I‐gel in clinical scenarios prioritizing maternal and fetal stability. However, this study was conducted in a single‐center setting with a relatively homogeneous patient population, potentially introducing selection bias and limiting generalizability to broader ICU environments like those in our msPDT cohort.

Cools‐Lartigue et al. [19] highlighted the evolution of minimally invasive tracheostomy techniques, emphasizing innovations like bronchoscopic guidance to reduce complications. Similarly, studies in laparoscopic and general anesthesia settings reinforce the reliability of SADs like I‐gel and LMA ProSeal, which optimize ventilation stability and minimize invasiveness; critical features in msPDT [20, 21, 22].

\Although our results favor SADs for procedural efficiency in msPDT, conflicting evidence exists in scenarios where ETTs may outperform SADs. For example, in high‐airway‐pressure ventilation settings, such as patients with severe ARDS or those requiring peak inspiratory pressures exceeding 30 cm H₂O, ETTs provide a more secure seal and better tolerance for prolonged high‐pressure mechanical ventilation compared to SADs, which carry a higher risk of air leak or displacement [23, 24, 25]. Studies in analyses of airway devices during anesthesia indicate that the comparable risk of aspiration exists in such high‐pressure contexts, but ETTs are often preferred to minimize potential complications, potentially making them preferable over SADs despite longer insertion times [26]. This underscores that while SADs like I‐gel and LMA excel in low‐to‐moderate pressure msPDT procedures, ETTs remain essential in select high‐risk cases, emphasizing the importance of individualized device selection based on patient‐specific ventilatory demands.

Device‐specific design is a known determinant of procedural suitability, and our findings reveal that theoretical benefits do not always translate clinically. Although second‐generation SADs like the LMA ProSeal are praised for sealing efficacy [27, 28], the curved design of the LarySeal led to tracheal displacement in our study. This increased the risk of para‐tracheal puncture and a 7.1% pneumothorax incidence, a mechanism we visualized via ultrasound, representing a novel contribution.

These results caution against the broad endorsement from some meta‐analyses, which may overestimate sealing efficacy due to single‐center bias and procedural variability [21]. Our study, though also single‐center, offers mitigating objective evidence through ultrasound‐guided anatomical analysis, highlighting the necessity of balancing leak pressure data with anatomical compatibility and calling for robust multicenter validations.

The stability of hemodynamic and oxygenation parameters across groups reinforces the safety of msPDT as a bedside procedure. However, the slight SpO₂ decline in the ETT group likely reflects challenges in maintaining tube position during tracheostomy, consistent with prior reports of ETT dislodgement.

Strengths of this study include the integration of ultrasound imaging to evaluate device‐induced anatomical changes, providing mechanistic insights into LarySeal's limitations.

Though this study has several limitations that should be considered when interpreting the results. First, the single‐center design at a university‐affiliated hospital in Iran may introduce institutional bias, limiting generalizability to other settings with varying patient demographics, equipment availability, or procedural expertise. Second, the non‐randomized group allocation based on intensivist preference could lead to selection bias, although baseline characteristics showed no significant differences. Third, the small sample size in the LarySeal group (*n* = 14) reduces statistical power for detecting rare complications and renders those findings exploratory, potentially overestimating risks like pneumothorax. Additionally, while ultrasound provided valuable anatomical insights, its subjective interpretation by operators could introduce variability, and we did not assess long‐term outcomes such as tracheal stenosis or device‐related infections beyond the immediate post‐procedure period. These weaknesses highlight the need for multicenter, randomized controlled trials with larger cohorts to validate our conclusions and refine airway management guidelines in msPDT.

Clinically, I‐gel and LMA emerge as preferred devices for msPDT due to their efficiency and minimal anatomical interference, whereas LarySeal should be reserved for scenarios where its sealing properties outweigh misalignment risks.

## 5 Conclusion

This study establishes I‐gel and LMA as superior to ETTs and LarySeal in msPDT, offering shorter procedural times, fewer complications, and enhanced anatomical compatibility. LarySeal's design‐related tracheal displacement underscores the importance of device‐specific evaluation. While these findings support the adoption of SADs in msPDT, larger trials are needed to confirm clinical recommendations, optimize airway management protocols, and refine clinical guidelines.

## Author Contributions

**Seyed MohammadReza Hashemian:** design and conceptualization, project administration, supervision, writing – review and editing. **Ameneh Jafari:** investigation, validation, writing – original draft, writing – review and editing. **Hamidreza Jamaati:** conceptualization, validation, resources. All authors have read and approved the final version of the manuscript and they believe that the manuscript represents honest work. Seyed MohammadReza Hashemian, the corresponding author, had full access to all the data in this study and takes complete responsibility for the integrity of the data and the accuracy of the data analysis. **Mehran Malekshoar:** visualization, project administration. **Majid Malekmohammad:** visualization, project administration. **Batoul Khoundabi:** data curation, formal analysis, writing – review and editing. **Hossein sadegh:** investigation, writing – original draft.

## Funding

The authors received no specific funding for this work.

## Conflicts of Interest

The authors declare no conflicts of interest.

## Transparency Statement

The lead author Seyed MohammadReza Hashemian affirms that this manuscript is an honest, accurate, and transparent account of the study being reported; that no important aspects of the study have been omitted; and that any discrepancies from the study as planned (and, if relevant, registered) have been explained.

## Data Availability

The data that support the findings of this study are available on request from the corresponding author. The data are not publicly available due to privacy or ethical restrictions.
